# Knowledge, Attitudes, and Practices of Family Physicians Regarding Pre-travel Counseling for Patients With Type 2 Diabetes in Riyadh, Saudi Arabia

**DOI:** 10.7759/cureus.89221

**Published:** 2025-08-01

**Authors:** Nasser A Aldawsari, Bandar Alanazi, Abdulmalik Alangari, Abdulkarim Almuhaydili, Fahad Albahili, Fahad Almazyad

**Affiliations:** 1 Family Medicine Academy, King Fahad Medical City, Riyadh Second Health Cluster, Riyadh, SAU; 2 Department of Family Medicine, King Abdullah bin Abdulaziz University Hospital, Riyadh, SAU

**Keywords:** diabetes knowledge, diabetes type 2, family medicine, patient counseling, pre-travel consultation

## Abstract

Background: Traveling has become an important part of Saudi people's lives. Those diagnosed with type 2 diabetes mellitus (T2DM) encounter various challenges during travel that can adversely affect glycemic control. Studies have highlighted critical gaps in the knowledge, attitudes, and practices (KAP) of family medicine physicians in Saudi Arabia regarding pre-travel counseling for patients with type 2 diabetes.

Objective: The study aimed to assess the KAP of family medicine physicians regarding pre-travel counseling for patients with T2DM.

Methodology: A cross-sectional study was conducted among family physicians working in primary healthcare centers and hospitals in Riyadh, Saudi Arabia, from December 2024 to June 2025. A convenience sampling technique was used to recruit participants. Data were collected through a validated self-administered questionnaire, and appropriate statistical tests were used to perform univariate analysis.

Results: A total of 277 family physicians from Riyadh participated in the study, comprising 52.3% male and 47.7% female physicians. The majority (74.0%) were 30 years or younger. Most participants were residents (62.5%), followed by consultants (19.1%) and registrars (18.4%). Regarding years of experience, 79.1% had 0-5 years of experience. The mean knowledge score was 2.04 (SD = 0.399), with 68.2% of physicians demonstrating knowledge about diabetes and travel. Higher knowledge scores were observed among consultants compared to registrars and residents. In practice, 54.9% estimated seeing 20-40 diabetes patients per week, but 60.6% reported that patients rarely asked for travel advice. Most consultations lasted 5-15 minutes (34.3%). Only 32.1% physicians were aware of travel safety recommendations, and 16.2% felt confident in adjusting insulin doses across time zones.

Conclusion: Collectively, our findings position Riyadh physicians within a broader regional pattern of moderate knowledge, supportive attitudes, and suboptimal practices regarding pre-travel diabetes care.

## Introduction

Traveling has become an important part of Saudi people's lives. People diagnosed with type 2 diabetes mellitus (T2DM) encounter various challenges while traveling that can have detrimental effects on glycemic control. However, more than half (57.9%) of primary healthcare physicians in Riyadh showed poor knowledge about pre-travel counseling for patients with type 2 diabetes. Poor knowledge was significantly associated with being younger, male, Saudi, a general primary healthcare provider, and having modest experience (0-5 years). Additionally, 47.5% of physicians disagreed with the importance of pre-travel counseling, a view more prevalent among older physicians with years of experience. Most of them (62.6%) also had poor practice scores, especially among younger, male, Saudi general practitioners [1]. Despite the increased outbound travel from Saudi Arabia, a study from the Eastern Province found that 76.8% of travelers had never sought pre-travel health consultations. Important barriers included perceived low risk (74.8%), lack of awareness (36.3%), time constraints (21.6%), and cost concerns (15.5%). Recommendations such as vaccination and disease prevention were deemed crucial by travelers [2].

In the Al Qassim region, nearly half of the physicians (49.2%) demonstrated fair knowledge about travel medicine. Positive attitudes and practices were observed among younger physicians with fewer years of experience and limited exposure to travelers. However, only about half engaged regularly with travelers, indicating a gap in knowledge and practice that needs to be addressed through continuing medical education programs [3]. Research from Qatar showed that primary care physicians' overall knowledge score about travel vaccines and malaria prophylaxis was 59.6%. Higher knowledge was significantly associated with middle age, medical degrees from non-Arab countries, prior training in travel medicine, and a high volume of travel consultations [4]. In the Jazan region, primary healthcare physicians had moderate knowledge and practice scores and encouraging attitudes toward pre-travel counseling for diabetic patients [5]. A study on blood sugar control among diabetic travelers showed hypoglycemic episodes in 8.6% of patients, which was significantly associated with crossing multiple time zones [6].

Another study from Riyadh indicated that only 23.7% of diabetic patients had been advised by their physicians on pre-travel precautions, while 20.7% encountered complications during their trips. Factors prompting consultations included patients' concerns about trip duration and risks, emphasizing the need for proactive physician-patient communication [7]. In Dammam and Qatif, diabetic travelers showed low awareness (67.4%) and inadequate practice (44.5%) regarding pre-travel counseling. Factors such as treatment type, gender, and previous travel history significantly influenced awareness and practice levels, suggesting that targeted educational interventions are required [8]. A survey of individuals with type 1 diabetes traveling long distances found that 74% reported experiencing increased hypoglycemia and/or hyperglycemia during overseas travel, and 22% had run out of insulin at some point during their trip. Issues highlighted included the lack of adequate resources addressing emergency protocols abroad [9]. In France, primary care physicians' effectiveness in pre-travel consultation was strongly associated with their motivation and proximity to vaccination centers. High knowledge scores were significantly correlated with the motivation level of physicians to practice travel medicine [10]. A census study from Scotland among diabetic insulin users revealed that around 10% encountered problems during travel, mainly hypoglycemia. Travelers desired improved and consistent advice, preferably accessible through clinic resources such as websites, highlighting the role of structured patient education to minimize travel-related health issues [11]. 

These studies collectively highlight critical gaps in the knowledge, attitudes, and practices (KAP) of primary healthcare physicians in Saudi Arabia regarding pre-travel counseling for patients with T2DM. Addressing these gaps through targeted educational interventions and standardized guidelines is essential to enhance diabetic patient safety and health outcomes during international travel. Given that the prevalence of T2DM in the Saudi population is assumed to be around 20%, with about 40% of them traveling internationally, this yields an approximate incidence of 8% of the general population with T2DM engaging in travel annually [7,12,13].

## Materials and methods

Study setting and sample size

A cross-sectional observational questionnaire-based study was conducted among family physicians working in primary healthcare centers and hospitals in Riyadh, Saudi Arabia, during the period from December 2024 to June 2025. The population number was 567, as estimated by the Ministry of Health (MOH) Statistical Yearbook in 2022 [14]. All family physicians working in either governmental primary healthcare centers or governmental hospitals were eligible for inclusion in the study. Family physicians working in the private sector or non-family physicians were excluded. A convenience sampling technique was employed. A standardized sample size equation, n = z^2^p(1−p)/e^2^, was used to calculate the sample size, with an assumed population proportion of 50%. Using a 95% confidence interval and a 5% margin of error, the sample size was estimated to be 230 and increased to 253 by adding 10% to compensate for a potential low response rate.

Data collection procedures and tools

A validated, self-administered questionnaire was utilized and sent electronically to family physicians in Riyadh, Saudi Arabia. The questionnaire was validated and used previously in a similar study conducted by Alduraibi et al. [1]. It contained a total of 24 questions divided into four sections. The first section gathered demographic information about the physicians (age, nationality, gender, specialty, educational degree, and years of medical experience after graduation). The second section assessed family physicians' knowledge about the travel of patients with diabetes (10 items), with responses categorized as 'yes', 'no', or 'do not know'. 'Yes' answers were assigned a score of 1, while 'no' and 'do not know' responses were assigned a score of 0. A total score was calculated for each participant and used for comparison purposes. The third section evaluated physicians' attitudes toward pre-travel counseling for diabetic patients (six items) using a five-point Likert scale ranging from 1 (strongly disagree) to 5 (strongly agree), with 'agree' assigned a score of 4, 'uncertain' a score of 3, and 'disagree' a score of 2. The overall attitude score was calculated, with higher scores indicating more positive attitudes. The fourth section provided a thorough evaluation of the practices of physicians in providing pre-travel counseling to patients (eight items). These were rated such that a higher score reflected more optimal practice.

Data management and analysis

Data were analyzed using IBM SPSS Statistics version 24.0 (IBM Corp., Armonk, USA). Appropriate statistical tests were used to perform the univariate analysis. The chi-square test was employed to analyze categorical data, which were displayed as numbers and percentages. Continuous data were assessed for normality using the Shapiro-Wilk test. Non-normally distributed data were presented as medians with interquartile ranges (25th-75th percentiles). Independent non-normally distributed continuous data were analyzed with the Mann-Whitney U test, while paired non-normally distributed continuous data were analyzed using Friedman’s two-way test. The F-test was used to analyze variance data. The t-test was utilized when comparing the means of two groups for quantitative (numerical) data. A p-value of ≤0.05 was considered statistically significant.

Ethical considerations

Study approval was obtained from the Institutional Review Board, Faculty of Medicine, King Fahad Medical City, Riyadh, Saudi Arabia (No. 24-691). Each participant was provided with informed consent before completing the questionnaire. In terms of copyright, permission for the survey used in the study was granted by the primary investigator of Alduraibi et al. [1]. The questionnaire was validated and used previously in a similar study conducted by Alduraibi et al. [1].

## Results

Table 1 represents the correlation coefficient for each statement by its factor for the questionnaire evaluating the KAP of family physicians toward pre-travel counseling of patients with type 2 diabetes in Riyadh, Saudi Arabia. The reliability of the questionnaire was measured using Cronbach's alpha correlation coefficient for knowledge and attitude factors of the questionnaire and for each statement of both factors. The correlations were very high, with an overall coefficient of 0.948, and all items had high correlation coefficients with their respective factors, statistically significant at the 0.05 level. On the other hand, the validity of the questionnaire was calculated using Cronbach's alpha and assessed through the split-half method, resulting in a value of 0.918, with statistical significance at the 0.05 level. The correlation results confirmed the validity of the questionnaire for application.

**Table 1:** Cronbach's alpha correlation coefficient for the questionnaire. ID: identity document

Factor	Statement	Cronbach's alpha	
Section 2: Knowledge	Patients with diabetes should be advised to carry medicines and carbohydrate-rich snacks in easily accessible bags while traveling	0.889	0.921
	Insulin can be stored in checked luggage	0.877	
	In air travel, patients with diabetes are advised not to inject insulin at takeoff	0.925	
	Traveling across more than five time zones requires insulin dose and frequency adjustment	0.896	
	Traveling across more than five time zones requires oral anti-hypoglycemic dose adjustment	0.832	
	Patients with diabetes traveling to the East region may need to increase their insulin dose	0.916	
	Patients with diabetes traveling to the West region may need to decrease their insulin dose	0.835	
	Extremes of hot or cold climate can affect how insulin and blood glucose are monitored in patients with diabetes while traveling	0.814	
	Is pre-travel vaccination important for patients with diabetes?	0.918	
	Patients with diabetes need to carry an ID that says they have diabetes while traveling abroad	0.829	
Section 3: Attitude	Pre-travel counseling for patients with diabetes is important	0.82	0.877
	The availability of an Arabic resource to increase the perception of health practice before, during, and after the trip is needed	0.839	
	If there is a trusted Arabic resource to increase the perception of health practice before, during, and after the trip, I will advise my patient to visit it	0.819	
	Seeking medical advice before travel will decrease the chances of getting sick during the trip	0.802	
	In Saudi Arabia, we lack travel medicine practice	0.752	
	Our society lacks the knowledge of the importance of travel medicine	0.821	
Total questionnaire		0.948	

As for sample description, a total of 277 family medicine physicians completed the questionnaire, exceeding the minimum estimated sample size of 253.

Table 2 shows the distribution of demographic characteristics of the sample participants. A total of 132 female physicians participated in the study, representing 47.7% of the total sample size, while 145 male physicians accounted for 52.3%. The mean age of participants was 30.1 years for the entire sample, with 205 physicians (74%) being younger than or equal to 30 years and 72 physicians (26%) older than 30. In terms of educational level, 173 physicians (62.5%) were residents, 51 (18.4%) were registrars, and 53 (19.1%) were consultants. Regarding years of experience, 219 physicians (79.1%) had less than five years of experience, 33 (11.9%) had 6-10 years of experience, 16 (5.8%) had 11-15 years of experience, and only nine physicians (3.2%) had more than 15 years of experience.

**Table 2:** Frequency distribution of the sample by sociodemographic variables.

Variable	Categories	Frequency (%)
Gender	Female	132 (47.7%)
	Male	145 (52.3%)
Age	≤30	205 (74%)
	>30	72 (26%)
Level of education	Consultant	53 (19.1%)
	Registrar	51 (18.4%)
	Resident	173 (62.5%)
Years of experience	0-5	219 (79.1%)
	6-10	33 (11.9%)
	11-15	16 (5.8%)
	>15	9 (3.2%)

From Table 3, we can see that 68.2% of the participants were aware of diabetes and travel, and 91.7% knew that patients with diabetes should be advised to carry medicines and carbohydrate-rich snacks in easily accessible bags while traveling. Furthermore, 87.5% were aware that patients with diabetes need to carry an identity document (ID) indicating their condition while traveling abroad, and 84.1% knew that patients with diabetes traveling to the East region may need to increase their insulin dose. Finally, only 52% of the participants were aware that patients with diabetes traveling to the West region may need to decrease their insulin dose. No statistically significant differences were observed between male and female participants or among participants with different years of experience. However, differences were noted in the level of education, particularly with consultants demonstrating the highest knowledge.

**Table 3:** Descriptive statistics for the knowledge factor. ID: identity document

Statement	Frequency (%)			Mean ± SD	%
	No	Neutral	Yes		
Patients with diabetes should be advised to carry medicines and carbohydrate-rich snacks in easily accessible bags while traveling	17 (6.1%)	26 (9.4%)	234 (84.5%)	2.75 ± 0.613	91.70
Insulin can be stored in checked luggage	148 (53.4%)	67 (24.2%)	62 (22.4%)	1.98 ± 0.683	66.06
In air travel, patients with diabetes are advised not to inject insulin at takeoff	37 (13.4%)	149 (53.8%)	91 (32.9%)	1.79 ± 0.909	59.69
Traveling across more than five time zones requires insulin dose and frequency adjustment	42 (15.2%)	142 (51.3%)	93 (33.6%)	1.82 ± 0.906	60.77
Traveling across more than five time zones requires oral anti-hypoglycemic dose adjustment	106 (38.3%)	129 (46.6%)	42 (15.2%)	1.69 ± 0.721	56.20
Patients with diabetes traveling to the West region may need to decrease their insulin dose	91 (32.9%)	154 (55.6%)	32 (11.6%)	1.56 ± 0.692	51.99
Extremes of hot or cold climate can affect how insulin and blood glucose are monitored in patients with diabetes while traveling	88 (31.8%)	153 (55.2%)	36 (13.0%)	1.58 ± 0.711	52.59
Is pre-travel vaccination important for patients with diabetes?	35 (12.6%)	101 (36.5%)	141 (50.9%)	2.14 ± 0.925	71.48
Patients with diabetes need to carry an ID that says they have diabetes while traveling abroad	12 (4.3%)	46 (16.6%)	219 (79.1%)	2.62 ± 0.754	87.48
Patients with diabetes traveling to the East region may need to increase their insulin dose	20 (7.2%)	56 (20.2%)	201 (72.6%)	2.52 ± 0.810	84.12
Knowledge factor				2.04 ± 0.399	68.2

Table 4 presents the descriptive statistics for the knowledge factor by educational degree. Consultant physicians had a mean knowledge score of 2.14 (SD = 0.38; n = 53), registrars had a mean score of 2.09 (SD = 0.41; n = 51), and residents had a mean of 2.00 (SD = 0.39; n = 173). The analysis of variance (ANOVA) test yielded an F-value of 13.18 with a p-value <0.001, indicating a statistically significant difference in knowledge scores among the three groups, with consultants scoring the highest.

**Table 4:** Test of the knowledge factor by education degree. Significant p-value ≤0.05. F-test was used for comparison.

Education degree	Frequency	Mean ± SD	F-test	P-value
Consultant	53	2.1434 ± 0.38904	13.182	0.000
Registrar	51	2.0941 ± 0.41829		
Resident	173	2.0023 ± 0.39310		

Table 5 outlines the knowledge factor categorized into three levels: poor (<1.6), moderate (1.6-2.3), and good (>2.3) to identify the impact of demographic variables on it. Gender, experience, and age showed no association with the knowledge factor. However, a statistically significant association was found between educational degree and knowledge level (p = 0.030), with residents showing the highest representation in the moderate knowledge category. Consultants and registrars exhibited relatively better knowledge compared to residents.

**Table 5:** Chi-square test of the knowledge factor by demographic variables. Significant p-value ≤0.05. Chi-square test was used for demographic variables comparison.

Variable	Knowledge categories (Frequency (%))				Chi-square	P-value
	Good	Moderate	Poor	Total		
Gender						
Female	35 (12.6%)	74 (26.7%)	23 (8.3%)	132 (47.7%)	2.599	0.273
Male	30 (10.8%)	95 (34.3%)	20 (7.2%)	145 (52.3%)		
Education degree						
Consultant	15 (5.4%)	32 (11.6%)	6 (2.2%)	53 (19.1%)	5.967	0.030
Registrar	17 (6.1%)	26 (9.4%)	8 (2.9%)	51 (18.4%)		
Resident	33 (11.9%)	111 (40.1%)	29 (10.5%)	173 (62.5%)		
Years of experience						
0-5	47 (17%)	139 (50.2%)	33 (11.9%)	219 (79.1%)	7.381	0.287
6-10	11 (4%)	15 (5.4%)	7 (2.5%)	33 (11.9%)		
11-15	3 (1.1%)	10 (3.6%)	3 (1.1%)	16 (5.8%)		
>15	4 (1.4%)	5 (1.8%)	0 (0%)	9 (3.2%)		
Age						
≤30	42 (15.2%)	131 (47.3%)	32 (11.6%)	205 (74%)	4.065	0.131
>30	23 (8.3%)	38 (13.7%)	11 (4%)	72 (26%)		

Table 6 indicates that 85.66% of the sample agreed with travel counseling for patients with diabetes, and 88.5% agreed that pre-travel counseling for these patients is important. Additionally, 88.8% of the sample agreed on the need for an Arabic resource to enhance the perception of health practices before, during, and after travel, while 87.8% believed that seeking medical advice before travel would decrease the chances of illness during the trip. Finally, 78.9% of the sample acknowledged a lack of travel medicine practice. Differences were noted between male and female participants regarding this factor, whereas the level of education and years of experience showed no differences in the attitude factor.

**Table 6:** Descriptive statistics for the attitude factor.

Statement	Frequency (%)					Mean ± SD	%
	Strongly disagree	Disagree	Neutral	Agree	Strongly agree		
Pre-travel counseling for patients with diabetes is important	11 (4%)	5 (1.8%)	22 (7.9%)	56 (20.2%)	183 (66.1%)	4.43 ± 0.996	88.52
The availability of an Arabic resource to increase the perception of health practice before, during, and after the trip is needed	9 (3.2%)	6 (2.2%)	28 (10.1%)	45 (16.2%)	189 (68.2%)	4.44 ± 0.986	88.81
If there is a trusted Arabic resource to increase the perception of health practice before, during, and after the trip, I will advise my patient to visit it	15 (5.4%)	9 (3.2%)	34 (12.3%)	43 (15.5%)	176 (63.5%)	4.29 ± 1.140	85.70
Seeking medical advice before travel will decrease the chances of getting sick during the trip	13 (4.7%)	4 (1.4%)	29 (10.5%)	46 (16.6%)	185 (66.8%)	4.39 ± 1.050	87.87
In Saudi Arabia, we lack travel medicine practice	14 (5.1%)	12 (4.3%)	68 (24.5%)	64 (23.1%)	119 (43%)	3.95 ± 1.142	78.92
Our society lacks the knowledge of the importance of travel medicine	12 (4.3%)	6 (2.2%)	45 (16.2%)	64 (23.1%)	150 (54.2%)	4.21 ± 1.069	84.12
Attitude factor						4.2828 ± 0.816	85.66

Table 7 presents the descriptive statistics for the attitude factor by gender. The average score for female participants (n = 132) was 4.17 with an SD of 0.987, while the mean for male participants (n = 145) reached 4.38 with an SD of 0.608. The t-test value reached 2.17 with a significance level of 0.031 (less than 0.05), indicating a statistically significant difference between male and female physicians, with male physicians scoring higher than female physicians in this factor. The chi-square test was also used to check the association between the attitude factor recoded from 1-5 to poor (less than 2.5), moderate (2.5-4.2), and good (4.2 or more) and the demographic variables of the study.

**Table 7:** Test of the attitude factor by gender. Significant p-value ≤0.05. T-test was used for comparison.

Gender	Frequency	Mean ± SD	T-test	P-value
Female	132	4.1717 ± 0.98718	2.175	0.031
Male	145	4.3839 ± 0.60805		

Table 8** **shows the distribution of attitude categories (good, moderate, poor) by demographic variables using the chi-square test. Among female physicians, 31.4% (n = 87) had a good attitude compared to 35.7% of male physicians (n = 99). Conversely, 11 female participants (4.0%) were in the poor attitude group compared to two male participants (0.7%). For education level, years of experience, and age, no significant associations were found with the attitude categories.

**Table 8:** Chi-square test of the attitude factor by demographic variables. Significant p-value ≤0.05. Chi-square test was used for demographic variables comparison.

Variable	Attitude categories (Frequency (%))				Chi-square	P-value
	Good	Moderate	Poor	Total		
Gender						
Female	87 (31.4%)	34 (12.3%)	11 (4.0%)	132 (47.7%)	7.690	0.021
Male	99 (35.7%)	44 (15.9%)	2 (0.7%)	145 (52.3%)		
Education degree						
Consultant	39 (14.1%)	10 (3.6%)	4 (1.4%)	53 (19.1%)	4.14	0.387
Registrar	32 (11.6%)	16 (5.8%)	3 (1.1%)	51 (18.4%)		
Resident	115 (41.5%)	52 (18.8%)	6 (2.2%)	173 (62.5%)		
Years of experience						
0-5	147 (53.1%)	63 (22.7%)	9 (3.2%)	219 (79.1%)	4.943	0.551
6-10	20 (7.2%)	11 (4.0%)	2 (0.7%)	33 (11.9%)		
11-15	11 (4.0%)	4 (1.4%)	1 (0.4%)	16 (5.8%)		
>15	8 (2.9%)	0 (0.0%)	1 (0.4%)	9 (3.2%)		
Age						
≤30	136 (49.1%)	61 (22.0%)	8 (2.9%)	205 (74.0%)	1.842	0.398
>30	50 (18.1%)	17 (6.1%)	5 (1.8%)	72 (26.0%)		

Table 9 shows that 54.9% of the sample estimated the number of patients with diabetes visiting the clinic per week to be between 20 and 40 patients, while 24.5% of the sample said that patients visited the diabetes clinic fewer than 20 times per week. Finally, 17.7% of the physicians in the sample estimated the number of patients with diabetes visiting the clinic to be more than 40 patients per week. 

**Table 9:** Percentage distribution of practice factor statements. ID: identity document

Statement	Categories	Frequency (%)
Estimated number of patients with diabetes who visit clinic per week	<20	68 (24.5 %)
	20-40	152 (54.9%)
	>40	49 (17.7%)
Number of patients with diabetes who ask for advice before a trip per month	>20	16 (5.8%)
	1-10	85 (30.7%)
	11-19	8 (2.9%)
If you counseled a patient with diabetes before traveling, how long did it take?	<5 minutes	52 (18.8%)
	>15 minutes	18 (6.5%)
	5-15 minutes	95 (34.3%)
	I did not counsel	110 (39.7%)
Recommended vaccines before travel	No	113 (40.8%)
	Yes	149 (53.8%)
Patients with diabetes trying to avoid travel	No	201 (72.6%)
	Yes	67 (24.2%)
Aware of travel safety recommendations for patients with diabetes	No	173 (62.5%)
	Yes	89 (32.1%)
Feel confident to adjust insulin dose for patients across time zones	No	226 (81.6%)
	Yes	45 (16.2%)
Patients mostly ask you about	I have never been counseled	127 (45.8%)
	Medication adjustment	83 (30.0%)
	Prescription	22 (7.9%)
	Vaccination	24 (8.7%)
	Diabetes ID and a letter	21 (7.6%)

While 168 physicians in the sample indicated that no patients asked for advice before traveling (60.6%), 85 physicians reported having only 1-10 cases of patients who asked for advice before a trip per month (30.7%). Only eight physicians said they had 11-19 cases of diabetes patients asking for advice (2.9%), and 16 physicians reported having more than 20 cases of diabetes patients seeking advice per month before travel (5.8%).

Regarding the time allotted for visits, 95 physicians had 5-15 minutes for each case (34.3%), 52 physicians had less than five minutes for each case (18.8%), and 18 physicians had more than 15 minutes for each case (6.5%). The remainder did not have any cases of diabetes. A total of 149 physicians (53.8%) recommended vaccines before travel for patients with diabetes, while 67 physicians (24.2%) encountered patients with diabetes who were attempting to avoid travel due to their illness. Additionally, 89 physicians (32.1%) reported awareness of travel safety recommendations for patients with diabetes, and 45 physicians felt confident about how to adjust insulin doses for patients traveling across multiple time zones (16.2%). About 83 physicians indicated that patients primarily inquired about medication adjustments (30%), 24 physicians said patients mostly asked about vaccinations (8.7%), and finally, 22 physicians reported that patients asked about prescriptions (7.9%).

## Discussion

Traveling has become an integral part of the Saudi population's life, notably among individuals managing chronic diseases such as T2DM. Traveling poses hazardous risks for patients with diabetes, including difficulties in maintaining blood glucose levels within the target range, potential hypoglycemic episodes, and challenges with medication compliance. Consequently, pre-travel counseling provided by healthcare professionals is important to minimize these risks and ensure patient safety. The objective of this study was to evaluate the KAP of family physicians in Riyadh regarding pre-travel counseling for patients with T2DM. Using a structured questionnaire, responses from 277 family physicians were analyzed. The study identified that while physicians agreed on the importance of pre-travel counseling, significant gaps in both their knowledge and practices were observed, particularly in areas such as adjusting insulin doses during travel and vaccination awareness.

In this study, 68.2% of physicians demonstrated adequate knowledge regarding diabetes management during travel, similar to the findings from Jazan, where moderate knowledge scores were similarly reported among primary healthcare physicians [5]. However, despite adequate knowledge, there was variability regarding specific management practices, particularly in adjusting insulin doses, echoing global challenges identified in the literature from Qatar, where overall knowledge about travel medicine was reported at 59.6% [4]. Physician attitudes towards pre-travel counseling were notably positive, with 88.5% acknowledging its importance. This aligns with prior regional studies indicating favorable attitudes but low practical implementation. For instance, research in the Al Qassim region revealed a similar level of attitude [3]. Our findings mirror data from the Eastern Province, where the majority of travelers never sought or received pre-travel health consultations [2]. Additionally, our findings identified that only 16.2% of physicians were confident in adjusting insulin doses for travelers across multiple time zones, a challenge also recognized in other studies. Notably, a cross-sectional study on diabetic travelers identified significant health complications caused by inadequate insulin management due to insufficient pre-travel consultation [6]. Furthermore, our research supports findings from Riyadh and Dammam, where similar knowledge-practice gaps indicate a critical need for educational interventions and standardized guidelines [1,8].

The clinical implications of this research are significant, emphasizing the need for structured, comprehensive pre-travel counseling as an integral part of type 2 diabetes management in family medicine settings. Implementing standardized pre-travel guidelines and integrating them into the routine care of type 2 diabetic patients could substantially reduce adverse health outcomes associated with travel. Moreover, promoting continuous medical education (CME) focused specifically on diabetes-related travel risks could greatly increase physician confidence and capability, leading to improved patient safety and overall clinical outcomes. Practically, this research highlights the need for increased public and professional knowledge regarding travel medicine among patients with T2DM. The development and dissemination of patient-oriented educational materials, ideally in Arabic, could significantly decrease travel-related health risks by empowering patients to proactively seek counseling before traveling. Healthcare policymakers and institutional leaders should prioritize embedding travel medicine training within existing medical curricula and CME programs to bridge the current knowledge-practice gaps.

A primary strength of this study is its robust methodological design, particularly the statistically justified sampling technique employed, which ensures a representative cross-section of family physicians from various demographic and professional backgrounds within Riyadh city. This enhances the generalizability of the study's outcomes to similar primary healthcare settings across Saudi Arabia. Another key strength of the research is the comprehensive approach adopted, examining multiple dimensions of physician preparedness (KAP), allowing for a generalized, holistic understanding of the factors influencing the provision of travel medicine. This approach can facilitate the identification of specific educational and practice gaps, thus offering targeted opportunities for intervention. The inclusion of demographic analyses further strengthens the applicability of findings in the study, providing actionable insights tailored for different physician groups.

Despite these strengths, certain limitations must be acknowledged. The cross-sectional nature of the study inherently limits the ability to infer causality or temporal relationships between physician characteristics and their KAP regarding pre-travel counseling. Moreover, reliance on self-reported data introduces potential response and recall biases, particularly regarding the reporting of counseling practices, which may not accurately reflect actual clinical behaviors. The study's geographic concentration in Riyadh potentially restricts the external validity of findings, particularly when considering rural or less urbanized regions. The exclusion of non-family physicians and specialists managing chronic diseases further narrows the scope of insights. These limitations parallel those reported in comparable regional studies, underscoring the need for broader multicenter investigations to better generalize findings across the Kingdom.

## Conclusions

Collectively, our findings position Riyadh physicians within a broader regional pattern of moderate knowledge, supportive attitudes, and suboptimal practices regarding pre‑travel diabetes care. Aligning CME, patient education, and system‑level prompts offers a pragmatic pathway to translate positive attitudes into effective counseling, thereby reducing travel‑related complications for the growing diabetic population.
